# Advanced Steel Materials: Recrystallization, Phase Transformation and Microstructure Analysis

**DOI:** 10.3390/ma18133080

**Published:** 2025-06-29

**Authors:** Toshio Ogawa

**Affiliations:** Department of Mechanical Engineering, Faculty of Engineering, Aichi Institute of Technology, 1247 Yachigusa, Yakusa-cho, Toyota 470-0392, Japan; ogawa.toshio@aitech.ac.jp; Tel.: +81-56-548-8121; Fax: +81-56-548-0277

Metals—such as steel—have a wide range of applications. A crucial aspect of material design is the precise control of recrystallization and phase transformation during manufacturing. The interaction between recrystallization and phase transformation plays a significant role in controlling the microstructure. The long history of research on the recrystallization and phase transformation of steel and other metals is well established [1,2,3]. Recently, in addition to experimental approaches, other methods such as modeling, simulation, high-dimensional analysis, and machine learning have gained attention [4,5,6]. These approaches have led to new and important findings. Therefore, this Special Issue focuses on the recrystallization and phase transformation of steel and other metals.

The first study analyzed the rolling and recrystallization textures of pure iron with different cold reduction ratios and cold-rolling directions. The texture evolution of pure iron after two-way cold-rolling and subsequent annealing was investigated through various experiments and machine learning techniques. As a result, effective two-way cold-rolling conditions for developing the Goss orientation were identified. Moreover, sensitivity analysis revealed that the annealing temperature was the dominant factor in the development of the Goss orientation. In future studies, inverse problem analysis should be performed to optimize the cold-rolling and annealing conditions for the development of each crystal orientation. In addition, the use of machine learning to analyze the microstructure and mechanical properties of metals is expected to grow in popularity.

The second study reports on the effects of the manufacturing process on the microstructure and mechanical properties of various types of steels. In particular, it investigates the effects of heat treatment conditions on the microstructure and mechanical properties of manganese steel and ultra-low-carbon steel. This study highlights the importance of proper microstructural control through heat treatment for the further development of steel properties. Another study examined the effects of thermomechanical conditions, high-temperature deformation, and cyclic ice plug deformation on the mechanical properties of steels. Offering a different perspective, a fourth study investigated hydrogen embrittlement in high-strength steel. Research on this topic is anticipated to become even more prevalent in the future.

Finally, this Special Issue features a study on the surface condition of steels, as well as a study on metals other than steel. All these studies are innovative, and further developments are expected in the future.

As mentioned above, the papers published in this Special Issue will make a prominent contribution to the future development of steel and other metals. I am eager to contribute, in whatever small way I can, to the advancement of steel and other metals.
